# Penam Sulfones and β-Lactamase Inhibition: SA2-13 and the Importance of the C2 Side Chain Length and Composition

**DOI:** 10.1371/journal.pone.0085892

**Published:** 2014-01-16

**Authors:** Elizabeth A. Rodkey, Marisa L. Winkler, Christopher R. Bethel, Sundar Ram Reddy Pagadala, John D. Buynak, Robert A. Bonomo, Focco van den Akker

**Affiliations:** 1 Department of Biochemistry, Case Western Reserve University, Cleveland, Ohio, United States of America; 2 Department of Molecular Biology and Microbiology, Case Western Reserve University, Cleveland, Ohio, United States of America; 3 Research Division, Louis Stokes Cleveland Veterans Affairs Medical Center, Cleveland, Ohio, United States of America; 4 Department of Chemistry, Southern Methodist University, Dallas, Texas, United States of America; 5 Department of Medicine, Case Western Reserve University, Cleveland, Ohio, United States of America; 6 Department of Pharmacology, Case Western Reserve University, Cleveland, Ohio, United States of America; Weizmann Institute of Science, Israel

## Abstract

β-Lactamases are the major reason β-lactam resistance is seen in Gram-negative bacteria. To combat this resistance mechanism, β-lactamase inhibitors are currently being developed. Presently, there are only three that are in clinical use (clavulanate, sulbactam and tazobactam). In order to address this important medical need, we explored a new inhibition strategy that takes advantage of a long-lived inhibitory *trans*-enamine intermediate. SA2-13 was previously synthesized and shown to have a lower *k*_react_ than tazobactam. We investigated here the importance of the carboxyl linker length and composition by synthesizing three analogs of SA2-13 (*PSR-4-157, PSR-4-155, and PSR-3-226*). All SA2-13 analogs yielded higher turnover numbers and *k*_react_ compared to SA2-13. We next demonstrated using protein crystallography that increasing the linker length by one carbon allowed for better capture of a *trans*-enamine intermediate; in contrast, this *trans*-enamine intermediate did not occur when the C2 linker length was decreased by one carbon. If the linker was altered by both shortening it and changing the carboxyl moiety into a neutral amide moiety, the stable *trans*-enamine intermediate in *wt* SHV-1 did not form; this intermediate could only be observed when a deacylation deficient E166A variant was studied. We subsequently studied SA2-13 against a relatively recently discovered inhibitor-resistant (IR) variant of SHV-1, SHV K234R. Despite the alteration in the mechanism of resistance due to the K→R change in this variant, SA2-13 was effective at inhibiting this IR enzyme and formed a *trans*-enamine inhibitory intermediate similar to the intermediate seen in the *wt* SHV-1 structure. Taken together, our data reveals that the C2 side chain linker length and composition profoundly affect the formation of the *trans*-enamine intermediate of penam sulfones. We also show that the design of SA2-13 derivatives offers promise against IR SHV β-lactamases that possess the K234R substitution.

## Introduction

**Figure 1:**
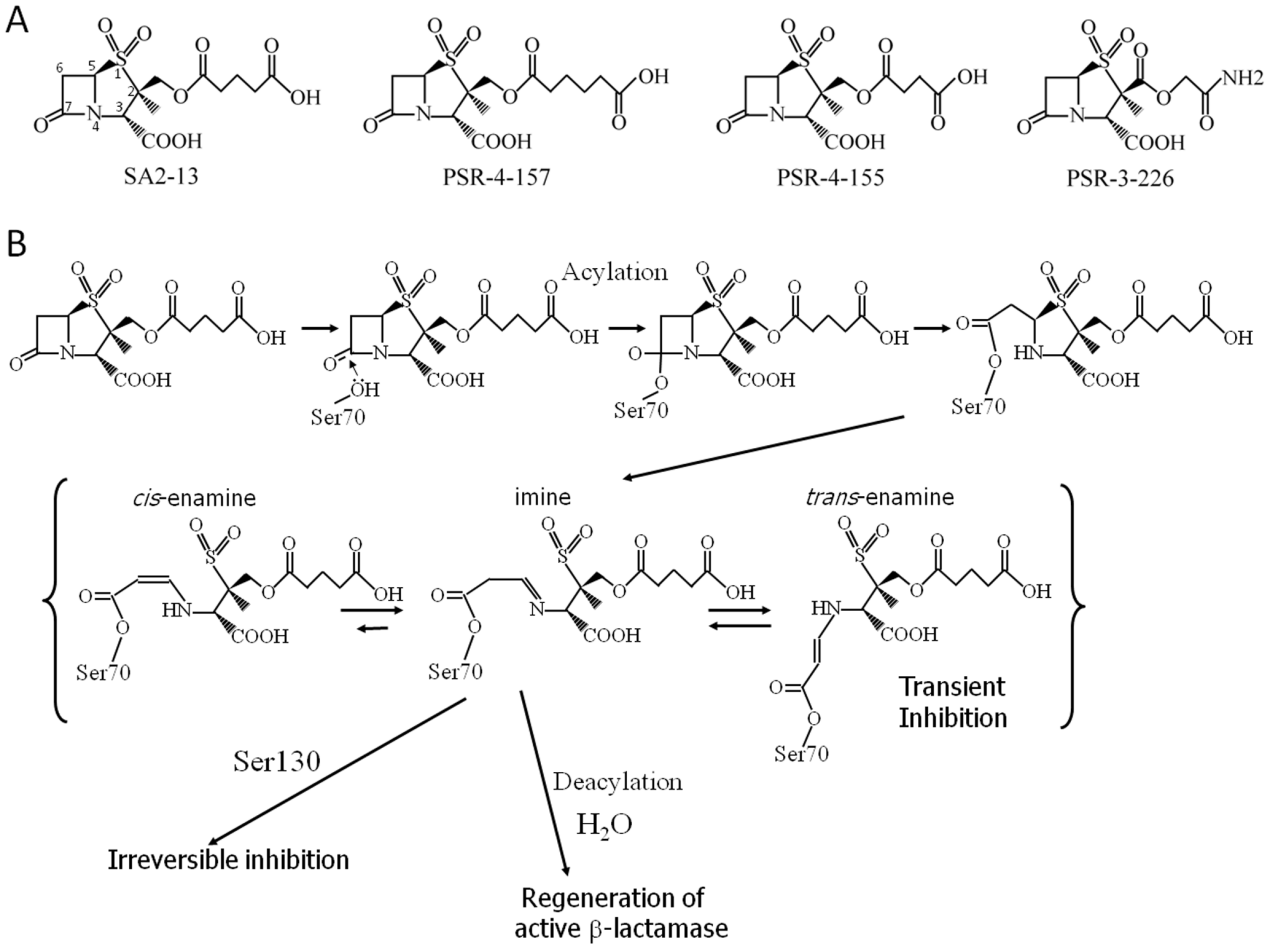
β-lactamase inhibitors. (A) Chemical structures for SA-2-13, PSR-4-157, PSR-4-155, and PSR-3-226. The ring atoms of SA2-13 are numbered to indicate the position of the C2 carboxyl linker and C3 carboxyl groups. (B) Proposed β-lactamase inhibition reaction scheme for SA2-13.

Bacteria harboring β-lactamase enzymes [E.C. 3.5.2.6] pose a significant threat to public health [1]. These enzymes prevent β-lactam antibiotics from reaching their intended target, the penicillin binding proteins (PBPs). To overcome this resistance determinant, β-lactamase inhibitors were introduced into the medical pharmacopeia. Currently, there are only three β-lactamase inhibitors clinically available (sulbactam, tazobactam and clavulanic acid, Figure 1). These inhibitors, when administered concomitantly with β-lactams, can aid in the eradication of infection by restoring the potency of the partner ß-lactam. Unfortunately, the inhibitors primarily inactivate class A β-lactamases [2], [3].

Sulbactam, tazobactam and clavulanic acid are mechanism-based inactivators as they share similarities with the β-lactam structure, the normal substrate for β-lactamases [3]. Similar to a substrate, these inhibitors can, after acylation at catalytic residue S70, undergo several inhibitory reaction pathways including fragmentation in the active site and covalently cross-linking at S130. This complex reaction chemistry leads to permanent inactivation thereby preventing further β-lactam hydrolysis (Figure 1B) [4]. In addition to the proposed irreversible inhibition mode, inhibitors can potentially also transiently inactivate the enzyme when the inhibitors tautomerize between *cis*- and *trans*-enamine conformations.

Regrettably, β-lactamases are being discovered with single or multiple amino acid substitutions that confer resistance to inactivation by these inhibitors, particularly clavulanic acid [3]. Presently, there are increasing numbers of new inhibitor resistant (IR) β-lactamases being discovered [5]. The appearance of these novel IR β-lactamase enzymes threatens the efficacy of existing β-lactam antibiotics spurring the clinical need to develop novel inhibitors that inactivate the target ß-lactamase by different mechanisms [3]. One such IR variant is the K234R variant which was first obtained in 1991 via a laboratory-generated K234R mutation in TEM [6]. In 2008, the K234R mutation was first identified in a clinical isolate harboring the SHV enzyme (SHV-56) which was found to be an IR β-lactamase [7] and subsequently observed in two additional IR variants: SHV-72 [8] and SHV-84 [9].

In order to design novel inhibitors, a number of different compounds with diverse modes of inhibition have been synthesized [3]. A promising approach recently taken is the stabilization of the *trans*-enamine species in order to extend the transient inhibition period [10], [11]. SA2-13, a penam sulfone, epitomizes this strategy [10]. We have previously shown that stabilizing the *trans*-enamine intermediate can successfully increase the longevity of the inhibitory intermediate as the *k*_react_ (a measurement of the recovery of active enzyme after inhibitor/enzyme incubation) of the designed SA2-13 inhibitor decreased by about 10-fold compared to tazobactam [10]. This lower *k*_react_ was achieved by replacing the triazolyl moiety of tazobactam with a carboxyl linker moiety targeting the carboxyl binding pocket of SHV-1 (see Figure 1A for chemical structure of SA2-13) such that the inhibitor would stabilize the *trans*-enamine conformation in a “U-shaped” conformation when it was covalently bound (see Figure 1B for the inhibition reaction scheme for SA2-13).

To gain additional insights into the functional characteristics of a designed carboxyl linker that optimizes *trans*-enamine stabilization and β-lactamase inhibition, we varied the carboxyl linker of potential inhibitors by adding one additional carbon to synthesize PSR-4-157. Next we removed a carbon to create PSR-4-155. Lastly, we changed the position of the ester bond, shortening the tail, and replaced the terminal carboxylate group with an amine group to result in PSR-3-226. In order to examine the consequences of these alterations, the derivatives were tested kinetically and structurally. Since it was the most effective inhibitor of class A β-lactamases in the series, we probed the inhibition efficacy of SA2-13 against the IR K234R variant of SHV to complement our previous studies that investigated the inhibition of the IR M69V variant and the IR S130G variant of SHV [11].

## Materials and Methods

### Synthesis of PSR-4-157, PSR-4-155, and PSR-3-226

**Figure 2:**
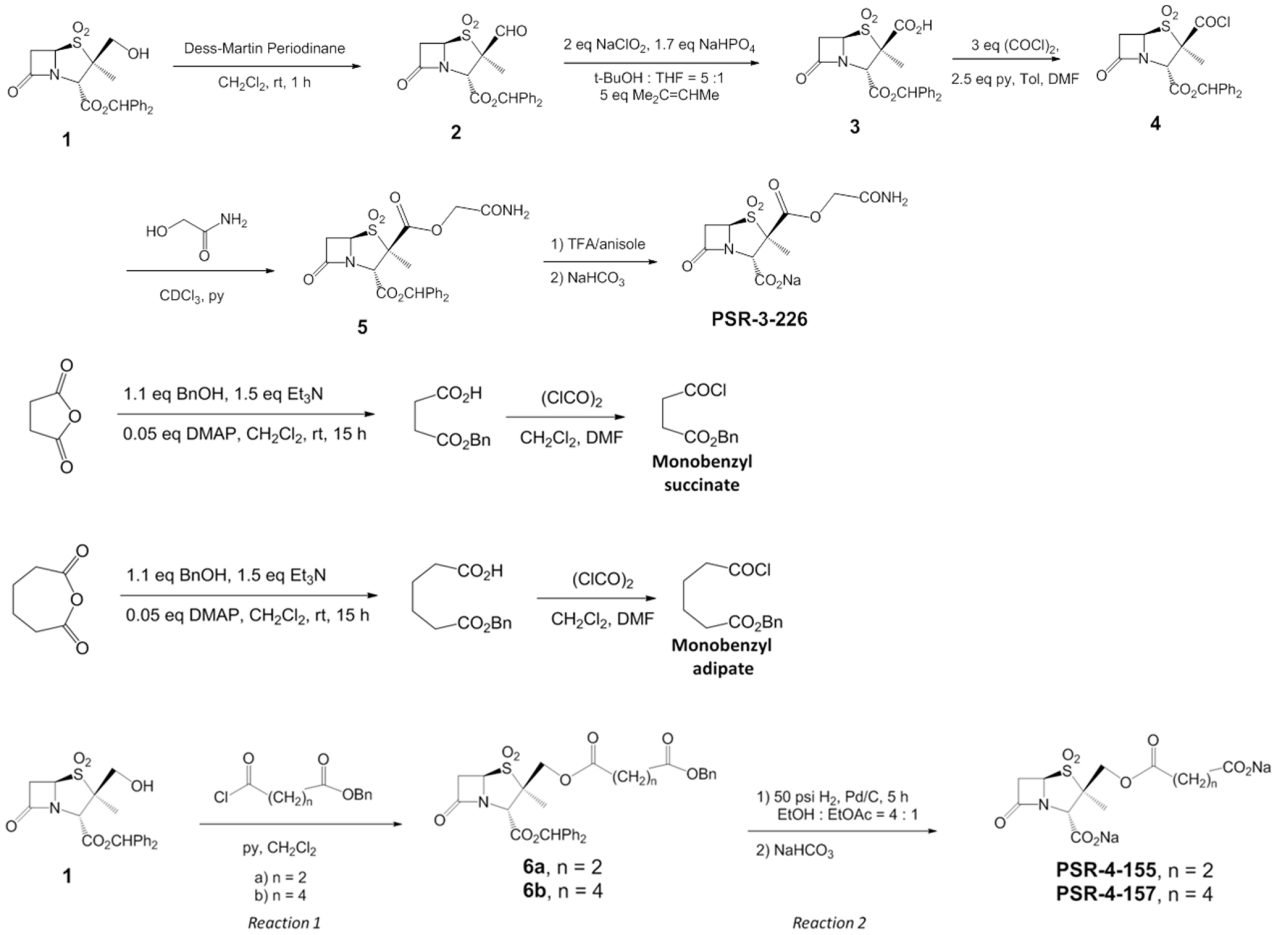
Synthesis of PSR-3-226, PSR-4-155, and PSR-4-157, the synthesis of SA2-13 is previously published [10].

PSR-3-226 was synthesized from intermediate **1** (Figure 2) as previously described [12]. PSR-4-155 and PSR-4-157 were prepared from monobenzyl succinate and monobenzyl adipate in a similar manner as for SA2-13 [10] (Figure 2). These two monobenzyl acids were converted to the respective acid chlorides, and reacted with intermediate **1** (labeled “reaction 1” in Figure 2). Subsequent deprotection (labeled “reaction 2” in Figure 2) produced the PSR-4-155 and PSR-4-157 inhibitors.

### Expression and purification

*wt* SHV-1, the deacylation deficient mutant E166A SHV, and the IR SHV variant K234R were subcloned and transformed as described previously [13], [14]. Briefly, the *wt* and E166A SHV variant containing cells were lysed using a stringent periplasmic lysis protocol; the lysate was subjected to preparative isoelectric focusing (pIEF) [15], followed by combining the nitrocefin positive fractions and loading them onto a Superdex75 size-exclusion column (GE LifeSciences). Two different protocols were followed for the IR K234R SHV variant purification as previously described [14].

For protein crystallization, the IR SHV K234R variant was subcloned into pET24a+ (Novagen) and expressed in OneShot BL21 Star (DE3) Chemically Competent *Escherichia coli* cells (Invitrogen) (as previously described [14]). Cells were disrupted and protein was released using a microfluidizer; the protein was purified to greater than 90% purity in a two-step process similar to the *wt* and E166A variant involving pIEF followed by gel filtration using a Superdex75 column (GE LifeSciences). For enzyme kinetics and circular dichroism (CD), the SHV K234R β-lactamase gene was subcloned into pGEX-6P-2 (GE Healthcare Life Sciences) and expressed in Origami2 (DE3) chemically compenent *E. coli* cells (EMD Millipore). The bacterial cells were disrupted by freeze-thawing and protein was released by the addition of lysozyme. The protein was purified using a GSTrap FF column (GE Healthcare Life Sciences) and size-exclusion gel filtration chromatography; the GST tag was cleaved using PreScission protease (GE Healthcare Life Sciences) and the final purification step was performed using the GSTrap FF column a second time. Fractions containing β-lactamase were detected with nitrocefin (NCF), a chromogenic cephalosporin. The NCF positive fractions were assessed for purity by sodium dodecyl sulfate polyacrylamide gel electrophoresis (SDS-PAGE) analysis and found to be greater than 90% pure.

### Kinetic assays

**Figure 3:**
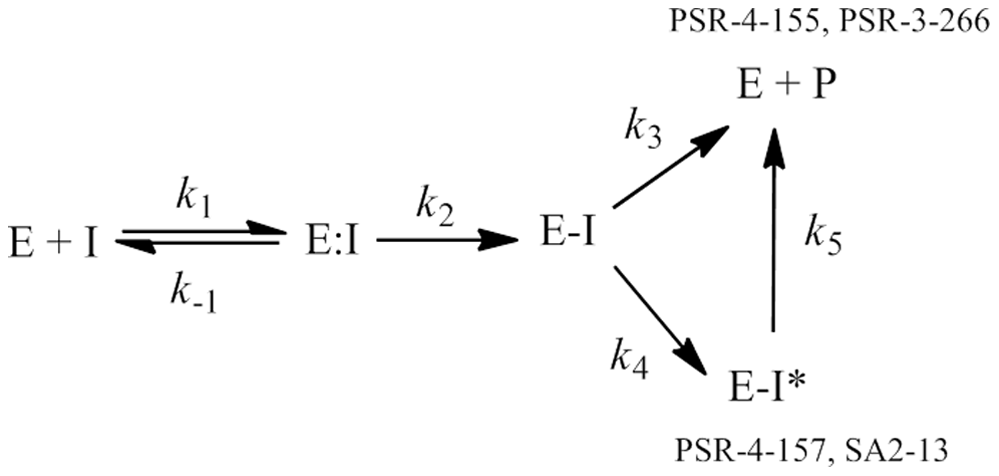
Reaction of enzyme (E) with inhibitor (I) leading to the formation of the Michaelis complex (E:I), acylated enzyme (E-I) and breakdown of the inhibitor to product (P) with regeneration of active enzyme.

In Figure 3 we represent a postulated mechanism for the behavior of SA2-13 and its three derivatives under study against SHV-1.

The *K*_i_ of the preacylation enzyme-inhibitor complex was calculated at room temperature (25°C) using an Agilent 8453 diode array spectrophotometer (Agilent Technologies) in a direct competition assay using 100 µM nitrocefin (BD Biosciences) (Δε_482_ = 17400 M^−1^cm^−1^), 3.5 nM enzyme and corrected for the nitrocefin affinity using equation 1:
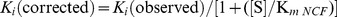
(1)where *K_m NCF_* is 20 µM for SHV-1 and *K*_m NCF_ is 16 µM for SHV K234R. Measurements were performed using glass cuvettes with 1 mL final volume in 1 mM phosphate-buffered saline (PBS) at pH 7.4 as previously described [14]. The IC_50_ was measured by pre-incubating the enzyme and inhibitor at 25°C and then measuring the inhibitor concentration leading to a 50% reduction in nitrocefin hydrolysis as previously described [16].

*k_inact_* (the first-order rate constant of inactivation) was determined by monitoring the inactivation of the enzyme by increasing concentrations of inhibitor over a time course using 21 nM of enzyme and 100 µM of nitrocefin according to a previously published method [17]. The *k*_obs_ determined using Origin (OriginLab Corporation) to plot the reciprocal of the ordinate of the intersection of the straight lines obtained from the initial velocity of inhibition, *v*_o_, and the final velocity of inhibition, v*_f_.* Each *k*_obs_ was plotted against the inhibitor concentration [I] and fit to equation 2 to calculate *k*_inact_ and *k*_inact_/*K*_I_. 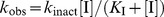
(2)


Determination of *k*_obs react_ (the apparent first-order rate constant for reactivation of the β-lactamase) was carried out by mixing 90:1 inhibitor:enzyme in a total volume of 80 µL, incubating the mixture for 30 min at room temperature in order to obtain a combination of inactive enzyme and enzyme-bound *trans*-enamine according to a previously published method [10]. Then, unbound inhibitor was washed out with a Macro Spin Column G10 (The Nest Group). A competition assay was performed with 100 µM of nitrocefin and a 5 µL aliquot of the incubated mixture at three different time points (5, 30, and 1440 min) to determine the reactivation of the enzyme. Tazobactam kinetic profiles are included for comparison. EnzFitter™ (Biosoft) and Microsoft Excel were used to analyze the data.

The partition ratio (turnover number, *t*_n_ = *k*_cat_/*k*_inact_) or the partitioning of the initial enzyme-inhibitor complex between hydrolysis and enzyme inactivation was measured by incubating fixed enzyme concentrations with increasing amounts of inhibitor over a 24 hour period in a total volume of 100 µL as described [18]. After 24 hours, a 20 µL aliquot of the incubation mix was removed and the steady-state hydrolysis of nitrocefin was measured and compared to an uninhibited control. The *t*_n_ was determined as the inhibitor proportion that led to a loss of >90% of nitrocefin hydrolysis by the enzyme.

Antimicrobial susceptibility testing involving bacteria expressing SHV-1 and AmpC PDC-3 is presented in Table S1. In brief, paper discs (6 mm diameter) were soaked with 10 µg inhibitor and 10 µg of ampicillin or cephalothin. Discs were placed on Mueller-Hinton (M-H) agar plates plated with 10^4^–10^5^ CFUs of *Escherichia coli* DH10B harboring SHV-1 or PDC-3 or *E. coli* ATCC 35218 containing TEM-1. Measured zone clearing diameters were used to determine susceptibility. Tazobactam results are included for comparison.

Circular dichroism (CD) was carried out on the SHV-1 and the SHV K234R proteins with and without SA2-13 and this is presented in Figure S1. In short, CD was performed on a JASCO J-815 spectrometer with a Peltier-effect temperature controller (GE Healthcare) as previously described [14]. Quartz cells with a 0.1 cm pathlength were used for experiments (Hellma). Thermal denaturation was performed from 22–72°C with a heating rate of 2°C/min and raw data was corrected for the fraction of denatured protein (*f*_u_). The collected data at 208 nm was utilized to calculate a melting temperature (*T*_m_).

### Crystallization and soaking

Crystals of *wt* SHV-1, E166A SHV, and K234R SHV were grown as described previously [10], [14]. The purified proteins were concentrated to 5 mg/ml using a 10K MWCO centrifugal concentrator (Amicon). Cymal-6 (final concentration 0.56 mM, Hampton Research) was added to the protein solution to grow crystals by sitting-drop vapor diffusion in 21–30% PEG6000 and 0.1 M HEPES pH 6.8–7.8.

The crystals were soaked in mother liquor containing 50 mM inhibitor for 30 minutes prior to being briefly transferred to a cryo-protectant solution consisting of mother liquor supplemented with inhibitor and 20–25% 2-methyl-2,4-pentanediol cryo-protectant. Inhibitors PSR-4-157 and PSR-4-155 were soaked into *wt* SHV-1 crystals; PSR-3-226 was soaked into crystals of the deacylation deficient E166A SHV variant as soaking this compound in *wt* SHV-1 crystals was not successful to crystallographically capture a stable intermediate (described in detail in Results and Discussion). The K234R SHV crystals were used for soaking experiments with 50 mM SA2-13 for 30 min. The crystals were subsequently flash frozen in liquid nitrogen prior to data collection.

### Data collection and refinement

Data was collected on beamline BL9-2 at Stanford Synchrotron Radiation Lightsource (SSRL) on a MAR-325 CCD detector. Integration and scaling was carried out using HKL2000 [19] and data statistics are shown in Table 1. The structures were solved using isomorphous replacement with the apo *wt* SHV-1 structure (PDB ID: 1SHV). The structure was refined using Refmac5 [20] in the CCP4 suite. COOT [21] was used for model building. Cymal-6 detergent molecules were observed in their established binding sites [22] and were included in refinement. After initial refinement of the protein and inclusion of the crystallant Cymal-6 and water molecules, difference electron density indicated the presence of covalently bound inhibitors PSR-4-157, PSR-3-226, and SA2-13 in their respective datasets. PSR-3-226 was modeled only as a partial inhibitor structure as the electron density did not reveal the entire inhibitor. The PSR-4-157 structure also contains an additional PSR-4-157 fragment that was non-covalently bound and distant from the active site (Figure S2).

**Table 1:** Data collection and refinement statistics.

Parameter	PSR-4-157 SHV-1	PSR-3-226 E166A SHV	SA2-13 K234R SHV
*Data Collection Statistics*			
Space Group	P2_1_2_1_2_1_	P2_1_2_1_2_1_	P2_1_2_1_2_1_
Cell Dimensions			
a, b, c (Å)	49.5, 55.2, 84.0	49.5, 55.3, 83.8	49.2,55.3,83.3
Wavelength (Å)	0.9795	0.9795	0.9795
Resolution (Å)	1.55	1.22	1.46
Highest resolution shell (Å)	1.55–1.61	1.22–1.26	1.46–1.51
R_sym_	0.051 (0.374)	0.039 (0.199)	0.051 (0.64)
I/σI	20.8 (2.6)	22.0 (6.2)	23.0 (2.0)
Completeness (%)	97.6 (90.2)	99.6 (99.0)	99.9 (100.0)
Redundancy	3.4 (3.0)	3.6 (3.5)	3.6 (3.5)
*Refinement Statistics*			
Resolution range (Å)	27.71−1.55	24.34−1.22	36.78−1.46
No. of Reflections	32086	65684	38131
R_work_/R_free_	0.167/0.196	0.137/0.161	0.178/0.214
No. of atoms (protein/ligand/water)	2123/93/170	2144/91/259	2119/67/161
RMSD			
Bond length (Å)	0.012	0.013	0.012
Bond angle (°)	1.49	1.70	1.46
Avg B-factors (Å^2^)			
protein	18.2	11.2	17.3
Ligands	25.7	16.3	26.8
Water	28.2	21.9	26.4
*Ramachandran plot statistics* (%)			
Core regions	91.3	90.9	90.0
Additionally allowed regions	8.2	8.7	10.0
Generously allowed regions	0.4	0.4	0.0
Disallowed regions	0.0	0.0	0.0

The linearized, *trans*-enamine conformation of each of these inhibitors was included in subsequent rounds of refinement. Stereochemistry library files for the inhibitors were obtained using the PRODRG server [23]. The *wt* SHV-1:PSR-4-157, E166A:PSR-3-226, and K234R:SA2-13 structures were refined to R/R_free_ values of 0.167/0.196, 0.137/0.161, and 0.178/0.214, respectively; the coordinates and structure factors have been deposited with the Protein Data Bank (PDB ID 4MBF, 4MBH, 4MBK, respectively).

## Results and Discussion

Three new SA2-13 penam sulfone analogs (PSR-4-157, PSR-4-155, and PSR-3-226) were synthesized, tested, and compared. Here, we present the kinetic profiles and structural investigations of these inhibitors. Antimicrobial susceptibility testing is presented in Table S1.

In these assays each of the inhibitors tested in combination with ampicillin demonstrated inhibitory effects on *E. coli* expressing *bla*_TEM-1_. These data indicate that the inhibitors are capable of both traversing the bacterial membranes and inhibiting a ß-lactamase in the periplasmic space. Inhibition was not observed for SHV-1 expressing cells by any of the inhibitors including tazobactam; this is likely a result of the high SHV-1 expression levels in the DH10B *E. coli* strain used (*bla*_SHV_ is under the control of 2 promoters in this construct, the *lacZ* and its natural promoter). Interestingly, PSR-3-226 had microbial activity versus the AmpC (PDC-3) of *Pseudomonas aeruginosa* (a class C β-lactamase) expressed in *E. coli* DH10B; the other inhibitors did not. This activity of this derivative versus an AmpC enzyme is a promising development.

### PSR-4-157 inhibition

We first characterized PSR-4-157 kinetically and the results are summarized in Table 2. Despite having a high *k*_react_ for SHV-1, PSR-4-157 demonstrated an overall inactivation efficiency (*k*_inact_/*K*_I_ = 0.10±0.02 µM^−1^s^−1^) comparable to tazobactam (*k*_inact_/*K*_I_ = 0.69±0.14 µM^−1^s^−1^). Compared to SA2-13 against SHV-1, PSR-4-157 had a 2-fold higher IC_50_. We were not able to obtain a *k*_obs react_ for SHV-1 using PSR-4-157 because full hydrolysis of nitrocefin occurred after the 30 minute incubation period.

**Table 2:** *Kinetic parameters* for SA-2-13 derivatives and tazobactam for comparison.

Inhibitor	IC_50_ (μM)	k_inact_ (s^−1^)	*k*_obs_ _react_ (s^−1^) 30 min	*K*_i_ (μM)	*K*_I_ (μM)	*k*_inact_/*K*_I_ (μM^−1^s^−1^)	*t*_n 24h_
**SA2-13**	0.081±0.007	0.07±0.01	0.004±0.001	1.7±0.1	0.19±0.01	0.37±0.06	120
**PSR-4-157**	0.16±0.01	0.10±0.01	NA†	1.7±0.1	1.0±0.2	0.10±0.02	>1500
**PSR-4-155**	0.44±0.04	0.11±0.01	>0.2	2.7±0.2	2.1±0.2	0.05±0.01	>1500
**PSR-3-226**	0.37±0.03	0.06±0.01	NA†	2.6±0.3	1.0±0.1	0.06±0.01	>1500
**tazobactam**	0.030±0.003	0.11±0.01	0.040±0.001	0.10±0.03	0.16±0.03	0.69±0.14	5

Some values were not applicable (NA) because steady-state had already been achieved when the assay with nitrocefin was started following the 30 min incubation. For these measurements recovery of full enzyme activity had already occurred.

**Figure 4:**
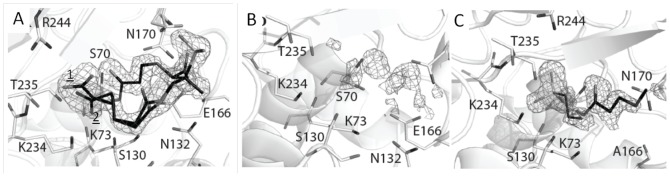
Electron density maps for inhibitors bound to SHV-1 β-lactamase. (A) |Fo|-|Fc| electron density difference map contoured at 3.0σ for PSR-4-157 prior to inclusion in refinement. The C2 carboxyl-tail linker was observed to be in two conformations. (B) |F_o_|-|F_c_| difference density for PSR-4-155 soaked *wt* SHV-1 soaked crystals revealing an absence of strong, interpretable inhibitor density. (C) |F_o_|-|F_c_| electron density difference map contoured at 3.0σ for PSR-3-226 prior to inclusion in refinement in the E166A SHV structure. Observed density reveals presence of a partially ordered PSR-3-226 fragment, right, and a HEPES buffer molecule fragment, left.

**Figure 5:**
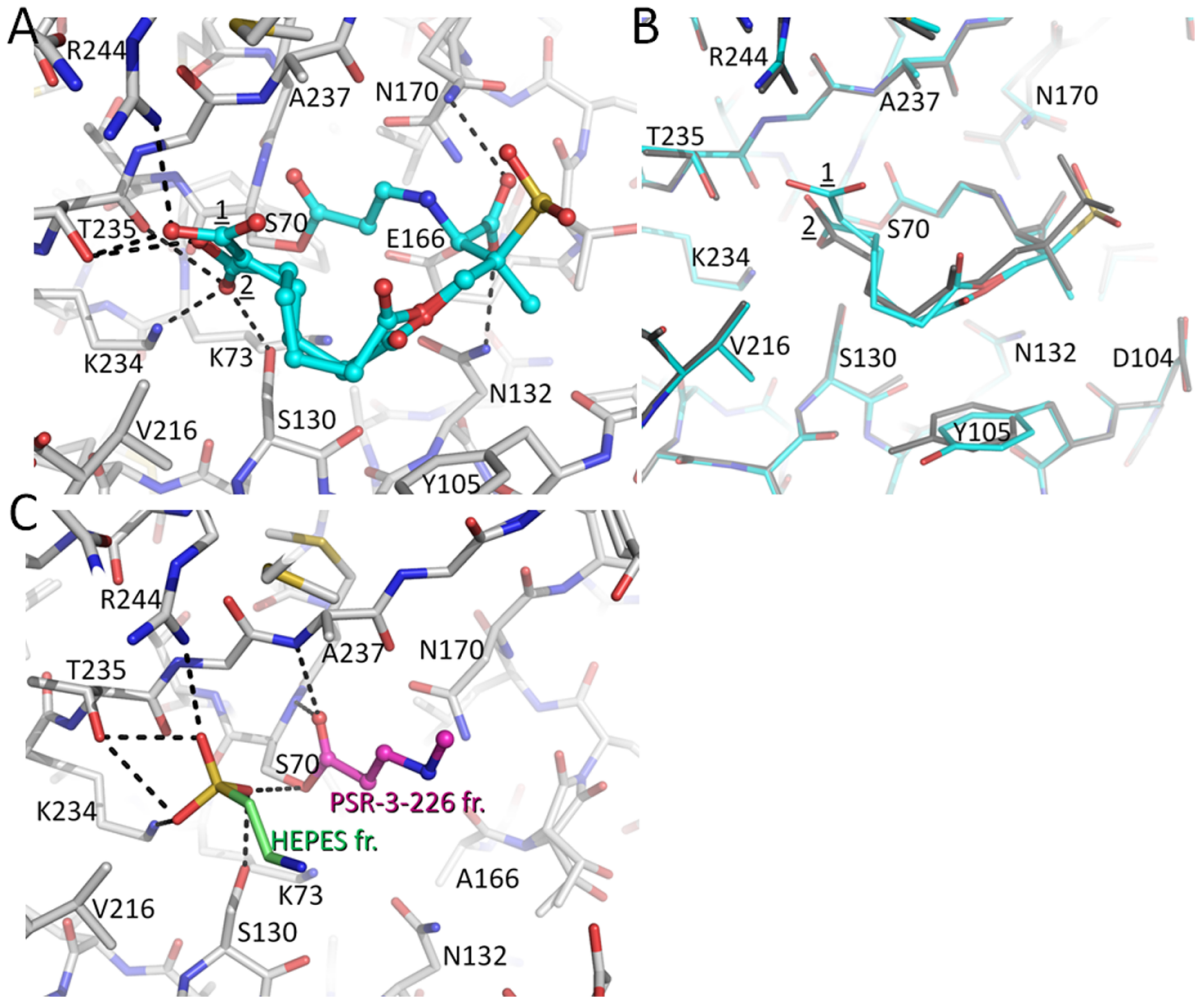
Interactions of the inhibitors in the active site. (A) Interactions of PSR-4-157 in the active site of *wt* SHV-1. The C2 carboxyl-tail linker of PSR-4-157 was observed in two conformations (labeled ‘1’ and ‘2’). Hydrogen bonds with the protein are indicated by dashed lines. (B) Superposition of PSR-4-157 (cyan) and SA2-13 (grey) when bound to *wt* SHV-1. The protein Cα atoms were used for the superpositioning. (C) Interactions of the ordered PSR-4-157 fragment in the active site of the deacylation deficient E166A SHV variant. Only 6 atoms of a covalently bonded PSR-3-226 intermediate could be built into the electron density (magenta carbon atoms); the nearby HEPES buffer molecule fragment (green carbon atoms) was also included in this figure.

By soaking *wt* SHV-1 crystals with 50 mM PSR-4-157 for 30 min we were able to determine the 1.55Å resolution acyl-enzyme structure with PSR-4-157 bound (Figures 4 and 5). Fo-Fc difference density revealed the presence of the inhibitor in the active site which was refined to an overall occupancy of 80% (Figure 4). The inhibitor was covalently bonded to S70 in a *trans*-enamine conformation with a torsion angle of 177° (Figure 5). For comparison, the *trans*-enamine torsion angles for SA2-13 bound to *wt* SHV-1 and the R164S/H variants are 166° [10] and 180° [24], respectively. This indicates that adding one extra carbon or being able to displace the Ω-loop as in the R164S/H variants, allows the *trans*-enamine bond of SA2-13-like compounds to adopt a torsion angle closer to (stereo-chemical) ideality (i.e. 180°).

The “tail” of PSR-4-157 adopts two conformations in the active site; the major conformation (60% occupancy) has its C2 carboxylate moiety shifted towards R244 whereas the (minor) second conformation (20% occupancy) positioned this C2 carboxylate in a similar position as the carboxylate of SA2-13 when bound to SHV-1 (PDB ID: 2H5S)(Figure 5A). The variation in C2 tail conformation is not unexpected as the longer C2 linker allows for some flexibility in this region. The first C2 carboxylate linker conformation brings the carboxylate within hydrogen bonding distance of R244, S130, T235, whereas the second conformation brings this moiety within hydrogen bonding distance of S130, K234 and T235. Additional interactions of the inhibitor involve the C3 carboxylate group which is within hydrogen bonding distance of N132 and one of the two N170 conformations.

Binding of PSR-4-157 in the active site of SHV-1 did not affect the overall structure of the enzyme as an all Cα superposition with the *wt* high resolution apo SHV-1 structure (PDB ID: 4FH4) yielded a low root mean square deviation (r.m.s.d.) of 0.21Å. Similarly, a superposition with the *wt* SHV-1/SA2-13 complex structure (PDB ID: 2H5S) yielded a low r.m.s.d. of 0.19Å for all Cα atoms. Minimal conformational changes within the active site are noted when comparing these structures (Figure 5B). The side chain of residue Y105 was slightly rotated (Figure 5B). Note, that all but one of the active site residues holds a single conformation; the exception is N170 which has two conformations at 50/50% occupancy (Figure 5B). One of these conformations was the canonical conformation seen in apo SHV-1 (PDB ID: 4FH4) oriented towards the deacylation water pocket; the other N170 conformer was oriented towards the inhibitor's C3 carboxylate group. This orientation of N170 was also previously observed in the *wt* SHV-1/SA2-13 complex structure [10] and was postulated to result in partial deacylation water occupancy as evidenced by weak Fo-Fc density for the deacylation water position.

### PSR-4-155 inhibition

PSR-4-155 has a carboxyl-tail linker that is one carbon atom shorter than in SA2-13 (Figure 1A). In kinetic analyses, PSR-4-155 appears to be a less potent inhibitor of SHV-1 compared to both SA2-13 and PSR-4-157 (higher *K*_i_ and higher IC_50_, Table 2). We attempted to capture PSR-4-155 crystallographically in the *wt* SHV-1 active site using soaking experiments (at 5, 10, and 30 min soaking times), without success. The 30 min soaking time which resulted in a 1.48Å resolution dataset showed possible density for just two atoms of a covalently bound carbonyl moiety potentially belonging to PSR-4-155 (Figure 4B). Beyond this density, no interpretable Fo-Fc density above 2.5σ was observed for the rest of the inhibitor. We suspect that the shortened C2 linker did not allow the C2 carboxyl moiety to reach the carboxyl binding pocket in the “U” shaped conformation as can occur for the longer SA2-13 and PSR-4-157. An inability to form the “U” shaped conformation prevents formation of the stable *trans*-enamine inhibitory complex. Because of the lack of clear PSR-4-155 density, the refinement was aborted.

Why weren't we able to capture a complex with PSR-4-155? The different crystallographic behavior of PSR-4-155 compared to the stable *trans*-enamine forming PSR-4-157 and SA2-13 inhibitors may be consistent with the measured kinetic properties. Upon analysis, SA2-13 is a better inhibitor compared to PSR-4-155 and PSR-4-157 with lower *k*_obs react_, *t*_n_, IC_50_, and *K_I_* values, and a higher *k*_inact_/*K_I_* (Table 2). In contrast, the kinetic differences between PSR-4-155 and PSR-4-157 are more modest. Nevertheless, PSR-4-155 appears less potent as a SHV-1 inhibitor compared to PSR-4-157 since its IC_50_ value is ∼3.5 times higher, its *K_i_* and *K_I_* values are almost 2-fold higher, and its *k_inact_/K_I_* is 2-fold lower compared to PSR-4-157. These kinetic differences may be one reason that PSR-4-155 is unable to form a stable crystallographically observable *trans*-enamine with SHV-1. In addition, it is still possible that some fraction of the active sites of SHV-1 soaked with PSR-4-155 contain a *trans*-enamine intermediate, with <0.2 occupancy that would probably not be crystallographically observable at 1.48Å resolution.

### PSR-3-226 inhibition

PSR-3-226 differs from SA2-13 in its C2 tail length, composition, and charge (Figure 1A). The kinetic parameters determined against SHV-1 are summarized in Table 2. PSR-3-226 was found to behave similar to PSR-4-157 with maximal differences of approximately 2-fold for *k*_inact_ and *K*_I_, Table 2.

PSR-3-226 was also not able to be trapped in the *wt* SHV-β-lactamase; we suspect that the tail of PSR-3-226 is not capable of forming a *trans*-enamine stabilizing U-shaped conformation. This prompted us to use the deacylation-deficient E166A variant of SHV-1 as we had done previously with difficult to capture compounds [25], [26]. Soaking E166A SHV-1 crystals for 30 minutes with PSR-3-226 (Figure 1A) yielded a 1.22Å resolution acyl-enzyme structure. Fo-Fc and 2Fo-Fc electron maps and subsequent crystallographic refinement indicated the presence of a covalently-bound partial PSR-3-226 fragment (refined at 100% occupancy) and a nearby HEPES buffer molecule fragment (refined at 80% occupancy; Figure 4C). A HEPES fragment situated at this position was previously also observed in the structure of the related apo SHV-2 β-lactamase [27]. The PSR-3-226 inhibitor fragment, which consists of six atoms, was covalently bound to S70 in a *trans*-enamine conformation (torsion angle of 175°) (Figure 5C). The fragment superimposed well with SA2-13 in the *wt* SHV-1 active site (PDB ID: 2H5S) and the HEPES buffer fragment occupies the same pocket as the C2 carboxylate of SA2-13. Since only six atoms of the inhibitor fragment were able to be resolved, there were few hydrogen bonds observed in the active site; the inhibitor carbonyl oxygen occupied the oxyanion hole, as in SA2-13- and PSR-4-157-bound SHV-1, within hydrogen bonding distance of the mainchain nitrogens of residues A237 and S70 (Figure 5C).

Like with previous E166A SHV inhibitor complexes, the PSR-3-226:E166A ß-lactamase appeared similar to both that of SA2-13:*wt* SHV-1 (2H5S) and *wt* apoSHV-1 (4FH4); superposition yielded an all-Cα atom r.m.s.d. of 0.17Å and 0.23Å, respectively [25], [26]. The PSR-3-226 structure showed few changes compared to the *wt* apoSHV-1 active site. The one exception was residue 166, which was substituted to an alanine from glutamate to capture the inhibitor intermediate. This E166A change resulted in a local distortion of the main chain for this residue similar to what we observed previously for deacylation-deficient E166A inhibitor complexes [25], [26].

We also previously determined the structure of PSR-3-226 bound to a different class A β-lactamase, KPC-2 [12]. When comparing this published PSR-3-226/KPC-2 structure to the PSR-3-226/SHV E166A structure, the *trans*-enamine moieties of the inhibitor are in a similar position. Interestingly, PSR-3-226 in both SHV-1 and KPC-2 is accompanied by a nearby buffer molecule interacting in the carboxyl binding pocket. In the SHV-1 complex, this buffer molecule is a HEPES fragment whereas in KPC-2, it is citrate [12]. Note that the KPC-2 complex was achieved with *wt* KPC-2 crystals, whereas the complex with SHV-1 was carried out with the deacylation deficient SHV-1 E166A variant. All atoms of PSR-3-226 could be resolved when bound to KPC-2 compared to only 6 atoms when bound to SHV-1. The *K*_i_ values for PSR-3-226 against SHV-1 and KPC-2 were found to be in the similar range (the *K*_i_ of PSR-3-266 against SHV-1 is 2.6±0.3 µM (Table 2) and the *K*_m_ of PSR-3-266 against KPC-2 is 3.8±0.4 µM, in this paper the defined *K*_i_ represents the *K*_m_ used in Ke *et. al*.[12]). Our inability to capture a stable complex crystallographically in *wt* SHV-1 crystals may have been because PSR-3-226 has a higher turnover number than SA2-13, tazobactam, or sulbactam with SHV-1, and PSR-3-226 likely cannot utilize the stabilizing K234 pocket interactions in SHV-1. In addition, as postulated for our inability to crystallographically capture *wt* SHV-1:PSR-4-155, the changes in the C2 tail length, composition, and charge of PSR-3-266 relative to SA2-13 may prevent formation of the “U”-shaped tail conformation and therefore prevent stable *trans*-enamine inhibition of *wt* SHV-1 by this compound. Like PSR-4-155, PSR-3-266 may form some *trans*-enamine intermediate at <0.2 occupancy, which is not crystallographically observable by our methods.

Our iterative analysis allows us to make some important observations: SA2-13 readily forms a stable, single conformation, 100% occupied, *trans*-enamine intermediate in *wt* SHV-1, which the three derivatives do not form. The explanation for the crystallographic differences between the three new inhibitors is likely subtle. PSR-4-157 has better inhibitory constants in most instances by only up to several-fold compared to PSR-4-155 and PSR-3-226 (Table 2). Perhaps the combination of these modest differences could allow PSR-4-157 to form a relatively stable *trans*-enamine intermediate (albeit with an occupancy of 0.8 and with its tail in two conformations) compared to the other inhibitors which could not form the *trans*-enamine intermediate in SHV-1.

From these observations, it is clear that *altering the length and composition of the carboxyl linker impacts the trans-enamine stability of penam sulfone inhibitors*. This property can be utilized in the design of future compounds optimized to inactivate these enzymes.

### SA2-13 inhibition of the IR SHV K234R variant

Intrigued by the long-lasting *trans*-enamine intermediate of SA2-13 and its favorable inhibitory properties of *wt* SHV-1, we extended our analysis to perform a kinetic and crystallographic characterization of SA2-13-inhibited K234R SHV to aid in the understanding of this class A IR variant. SA2-13 has the highest inactivation efficiency (*k*_inact_/*K*_I_) of our panel of inhibitors against *wt* SHV-1 (Table 2). *K_i_* values were determined for K234R SHV versus the inhibitors SA2-13 (1.4 µM), clavulanic acid (1.29 µM), and tazobactam (0.94 µM, Table 3). Also listed are the *K*_i_ values of these inhibitors against *wt* SHV-1. It is interesting to note that the *K*_i_ for SA2-13 is the least affected by the IR K234R substitution; only a 2-fold increase is observed relative to the *K*_i_ for *wt* SHV-1 compared to larger increases for the other inhibitors (the *K*_i_ for clavulanate is increased almost 8-fold and for sulbactam approximately 4.5-fold; see Table 3).

**Table 3:** *K_i_* values for *wt* SHV-1 and K234R SHV versus SA2-13, clavulanate, sulbactam, and tazobactam.

	*wt* SHV-1	SHV K234R
	*K*_i_ (μM)	*K*_i_ (μM)
SA2-13	1.4±0.1	2.1±0.2
Clavulanate	1.29±0.13	7.7±0.7
Sulbactam	1.57±0.16	4.6±0.5
Tazobactam	0.44±0.04	0.94±0.50

Previously, we determined the structure of the 1.08 Å IR apo K234R SHV variant [14]. This structure revealed that the R234 side chain adopts two conformations in the active site. The presence of the larger guanidinium group of arginine in K234R SHV leads to two alternate conformations of this group which likely affected the nearby S130 position, which was also found to have two conformations. We hypothesized that these changes in the carboxyl binding pocket region of the enzyme active site likely contribute to the IR phenotype of this SHV variant [14]. This was in agreement with an earlier study [8]. In addition, we had previously probed other IR SHV variants and investigated whether our designed *trans*-enamine stabilizing SA2-13 inhibitor is capable of inactivating these variants [11]. These IR SA2-13 inhibition studies showed that the S130G variant allowed for the *trans*-enamine intermediate to be formed but the M69V variant did not allow formation of this intermediate. We now extend these studies to IR K234R SHV.

**Figure 6:**
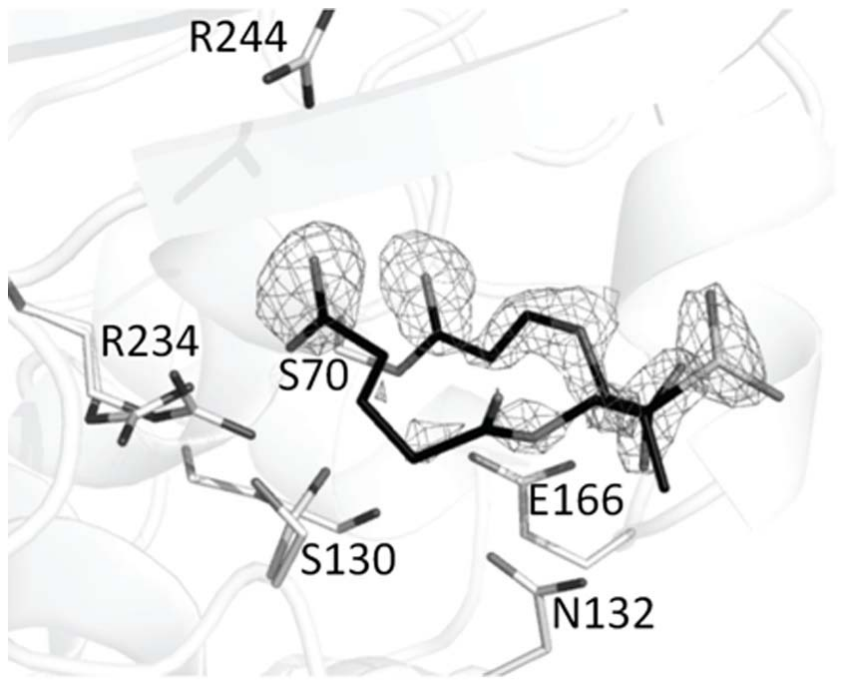
Electron density maps of SA2-13 bound in the K234R SHV active site. |F_o_|-|F_c_| electron difference density map is contoured at 3.0σ prior to inclusion of the inhibitor in refinement.

**Figure 7:**
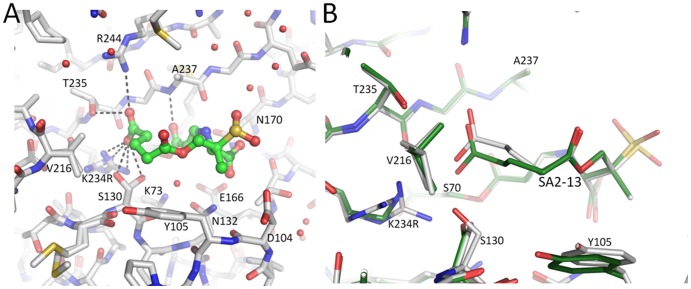
SA2-13 bound in the active site of IR K234R SHV. (A) Interactions of SA2-13 (green carbon ball-and-stick) covalently bound in the active site of K234R SHV (green carbon stick model). Hydrogen bonds are depicted as dashed lines. (B). Superpositioning of SA2-13 bound structures of *wt* SHV-1 (grey carbon atoms) and K234R SHV (green carbon atoms). The protein Cα atoms were used for the superpositioning.

After soaking and X-ray data collection, SA2-13 was observed to be covalently bound to S70 in the active site of K234R SHV in a *trans*-enamine conformation (torsion angle 179.7°, Figures 6 and 7). SA2-13 formed a number of important active site interactions. Most importantly, the C2 carboxylate participated in hydrogen bonding with R244, R234, T235 and both S130 conformations. Secondly, the C3 carboxylate of SA2-13 interacted with N132 via a hydrogen bond (Figure 7). When comparing the conformation of SA2-13 bound to *wt* SHV-1 and to the K234R SHV variant, SA2-13 adopts a similar conformation and position in both structures. There is however a minor shift observed in the C2 carboxyl moiety; this moiety has shifted 0.65Å away from the K234R substitution site (Figure 7B), likely as a consequence of needing to accommodate the larger guanidinium side chain of arginine at position 234. This adjustment in the position of the C2 carboxyl moiety of SA2-13 is similar, although not as large, as when SA2-13 is bound to IR S130G SHV [11].

The ability of the SA2-13 carboxyl moiety to shift to accommodate inhibition of different IR SHV variants indicates that SA2-13 is primed to adapt to different active site steric characteristics in the various IR enzymes. Although the C2 tail occupies roughly the same location in both the *wt* SHV-1 and K234R SHV structures, it appears as though the tail is slightly disordered in the active site of the latter structure as evidenced by its weaker density (Figure 6). This is likely due to the fact that R234 and S130 have alternate conformations in the K234R SHV structure which allows for more conformational heterogeneity in the C2 carboxyl tail region of SA2-13 than is occurring in the *wt* SHV-1 structure.

The K234R SHV protein itself has undergone little conformational changes upon SA2-13 binding as the r.m.s.d. of all Cα atoms between the apo and SA2-13 bound K234R SHV structures is only 0.18Å. The R234 residue also adopts two conformations, as in the apo K234R SHV structure. In addition, we also observed two conformations for residue S130 with 35/65% occupancy.

One difference between the apo- and SA2-13-bound K234R SHV structures involves the presence of the deacylation water. In the apo K234R SHV structure we observed one deacylation water [14] and in the SA2-13:SHV-1 structure we observed two partial occupancy waters in the deacylation water pocket [10]. However, in the K234R SHV:SA2-13 structure, the deacylation water is absent (even when Fo-Fc maps are contoured at 2.5σ). This lack of deacylation water may contribute to the inhibitory effect of SA2-13 on K234R SHV; the absence of this water is likely a consequence of the partial instability in the Ω-loop region as will be discussed next.

**Figure 8:**
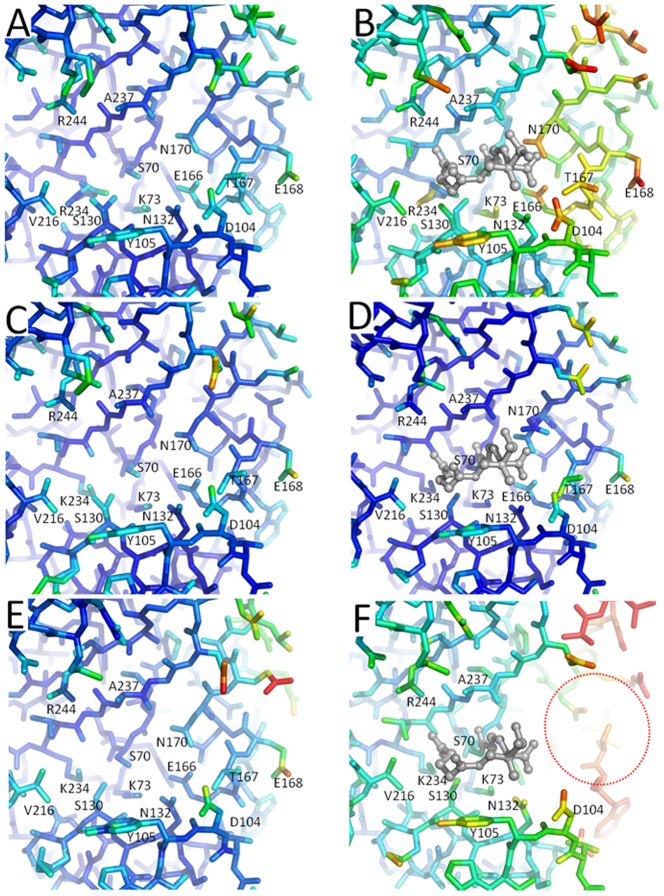
Protein disorder induced by SA2-13 binding. Temperature factors of protein atoms are represented by rainbow color-ramping from blue to red for temperature factors ranging from <5 to >40 Å^2^. Depicted are the structures of K234R SHV (A) and (B), wt SHV-1 (C) and (D), and R164S SHV (E) and (F). Uncomplexed structure are shown on the left (A), (C), and (E), whereas SA2-13 bound structures are on the right (B), (D), (F) with SA2-13 being depicted in grey ball-and-stick model. Completely disordered region not modeled in the SA2-13 bound R164S SHV structure is highlighted by red dashed oval.

Despite the limited structural changes in K234R SHV upon SA2-13 binding, there are also some interesting changes in protein mobility upon SA2-13 binding. These mobility changes are evident by the temperature factor differences of the structures (Figure 8). In the apo K234R SHV structure, the temperature factors of the proteins atoms are similar to the apo *wt* SHV-1 structure (Figure 8A and 8C). However, upon SA2-13 binding, a number of residues become more mobile in the K234R structure compared to the *wt* SHV-1 complex structure as evidenced by their temperature factor increases (Figure 8B and 8D). This was first noted by weaker 2Fo-Fc density for residues E166, N170, and the terminal atoms of the K73 side chain. The residues with increased temperature factors are predominantly located in the Ω-loop which encompasses residues 164–179. A similar, although more drastic increase in Ω-loop disorder upon SA2-13 binding was previously observed for the extended-spectrum β-lactamase R164S/H SHV variants [24]. In the R164S/H variant structures, the inhibitor caused a substantial portion of the Ω-loop to be disordered upon binding SA2-13 although in the absence of this inhibitor, this region was well-ordered (Figure 8E and 8F). We had hypothesized that this disorder was attributed to the electrostatic repulsion between the C2 carboxyl moiety of SA2-13 and the carboxylate of E166. In the *wt* SHV-1 protein, there is not a disorder transition upon SA2-13 binding, in contrast to mutants with a weakened Ω-loop such as the R164S/H variants. Interestingly, the observed disorder in the K234R SHV variant after SA2-13 binding (Figure 8B) suggests that the K234R substitution causes some destabilization of the Ω-loop. This is intriguing since the K234R residue is somewhat distant from the Ω-loop with the nearest atoms of this loop located approximately 8Å away. A possible explanation is an indirect effect by the larger sidechain and dual conformations of R234 leading to the dual conformations for the S130 residue. Both residues 234 and 130 are adjacent to K73 which also experiences increased temperature factors upon SA2-13 binding (Figure 8B). Since residue K73 makes a salt-bridge interaction with E166 of the Ω-loop, it is possible that the K234R substitution can exert an effect on the Ω-loop via changes in position and dynamics of residues S130 and K73 which are both located between residue K234 and the Ω-loop residue E166. Perhaps the SA2-13-mediated destabilized Ω-loop explains why the K234R SHV variant has a lower SA2-13 *K*_i_, compared to the other inhibitors (Table 3). A destabilized Ω-loop may prevent E166-mediated deacylation and turnover of SA2-13, which allows better inhibition of this IR enzyme by SA2-13. This would be reflected in a turnover number, not in the *K*_i_.

CD was done to further probe the effects of SA2-13 on the enzyme stability of both SHV-1 and SHV K234R (Figure S1). The overall UV spectrum of SHV K234R was unaffected by the presence of SA2-13, which indicates that this compound does not impact the overall secondary structure of the enzyme (Figure S1B). However, subtle changes were observed in the CD spectrum of SHV-1 after the addition of SA2-13 (Figure S1A). Additionally, the melting temperature, *T_m_*, of both enzymes was unaffected by the addition of SA2-13 (Figures S1C and S1D), which indicates that the stability of the proteins was not altered by the presence of SA2-13, in agreement with the lack of changes observed in the X-ray crystal structures of SHV-1 or SHV K234R upon complexing with SA2-13.

All considered, we find that the *trans*-enamine mode of inhibition using SA2-13 seems resistant to the K234R IR mutation in SHV-1. These insights could aid in future inhibitor design to exploit the *trans*-enamine mode of inhibition to counteract IR variants and novel β-lactamase enzymes.

## Conclusion

In summary, we show that efficacy of penam sulfone β-lactamase inhibitors designed with the SA2-13 scaffold rely heavily upon the C2 side chain length and composition. Based upon the knowledge that SA2-13 forms a long-lived *trans*-enamine intermediate, we show that the PSR-4-157 C2 chain length permits greater flexibility and allows more favorable interactions as compared to PSR-4-155 and PSR-3-226. However, all of these derivatives compare less favorably to SA2-13. We also showed that SA2-13 is able to inhibit the IR SHV K234R variant, likely as a result of its C2 side chain characteristics. Consequently, strategies to optimize *trans*-enamine intermediates could represent the basis of novel β-lactamase inhibitor design, particularly for IR β-lactamases.

## Supporting Information

Figure S1 **CD measurements of SHV-1 with and without SA2-13 (A) and SHV K234R with and without SA2-13 (B).** Thermal denaturation of apo-SHV-1 and SHV-1/MN-2-261 (54.2°C v 54.5°C, respectively) (C) and of apo-K234R and K234R/MN-2-261 (48.2°C v 48.7°C, respectively) (D)
(TIF)

Figure S2 **Electron density for second PSR-4-157 bound to surface of SHV-1.** Difference Fo-Fc density is calculated with the ligand removed from refinement (density is shown in blue contoured at 2.5σ level). Only density for the C2 carboxyl moiety of PSR-4-157 can be observed. The ligand is situated on the surface of the SHV-1 protein adjacent to cymal-6 located distant from the active site.
(TIF)

Table S1 **Antimicrobial disc assays**. These assays were performed with 10 µg ampicillin and 10 µg inhibitor. The size of the disc and the ampicillin zone size alone is 6 mm.
(DOCX)
