# Supplementary figures and images for: Penam Sulfones and β-Lactamase Inhibition: SA2-13 and the Importance of the C2 Side Chain Length and Composition
Source: PLoS One. 2014 Jan 16;9(1):e85892. doi: 10.1371/journal.pone.0085892 (PMC3894197; doi:10.1371/journal.pone.0085892)

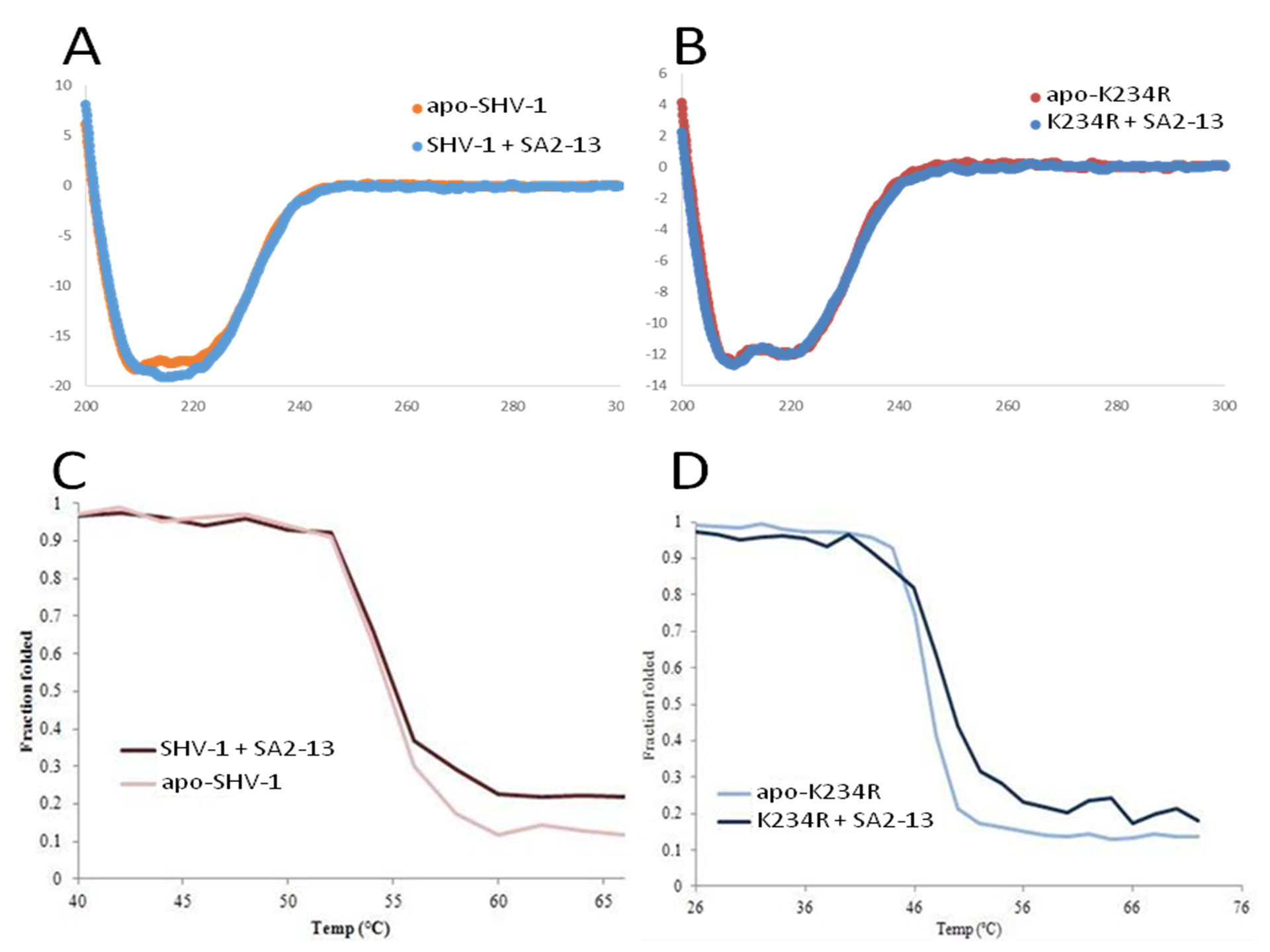

Supplement: Figure S1 — CD measurements of SHV-1 with and without SA2-13 (A) and SHV K234R with and without SA2-13 (B). Thermal denaturation of apo-SHV-1 and SHV-1/MN-2-261 (54.2°C v 54.5°C, respectively) (C) and of apo-K234R and K234R/MN-2-261 (48.2°C v 48.7°C, respectively) (D) (TIF) [file pone.0085892.s001.tif]

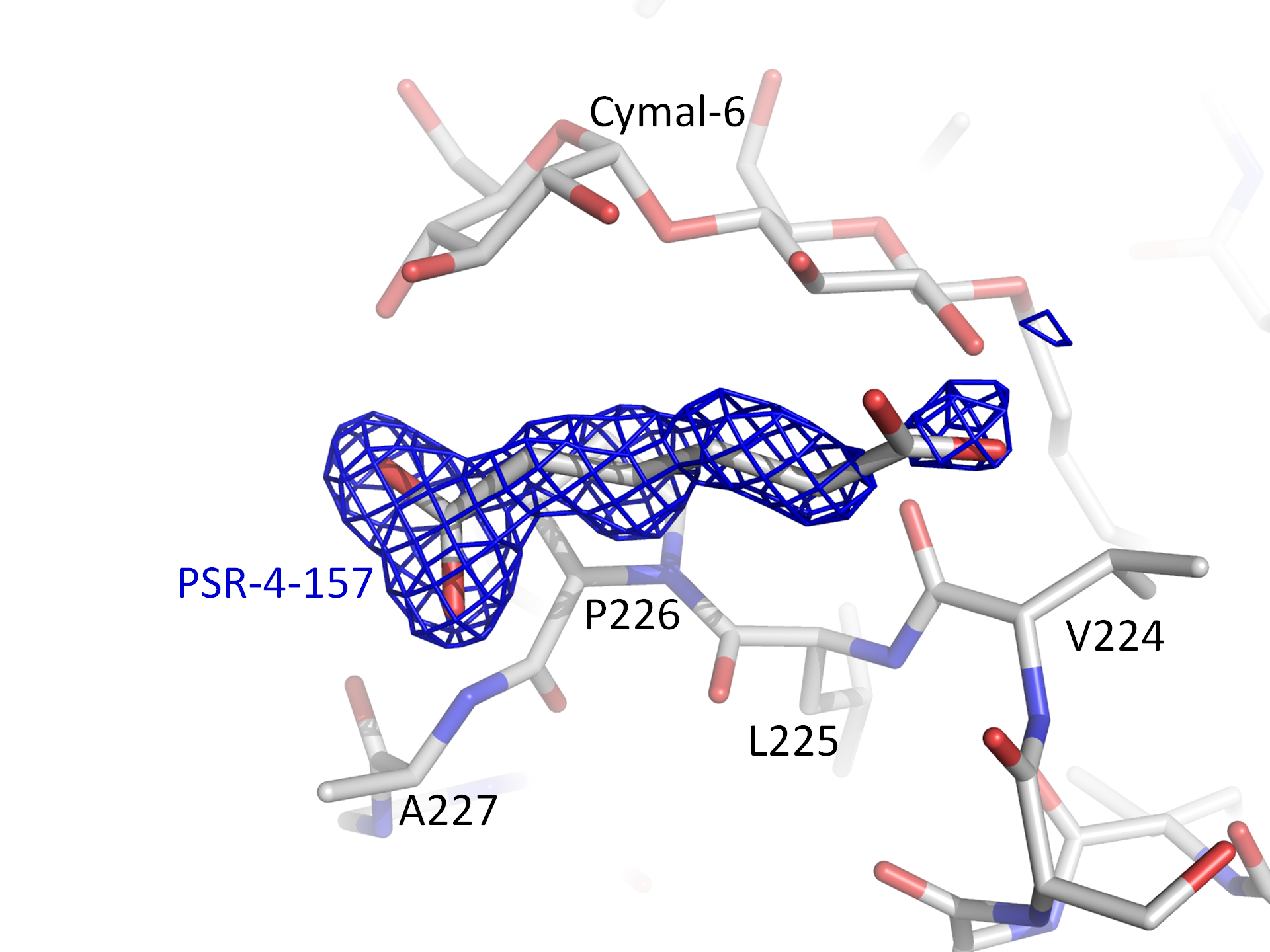

Supplement: Figure S2 — Electron density for second PSR-4-157 bound to surface of SHV-1. Difference Fo-Fc density is calculated with the ligand removed from refinement (density is shown in blue contoured at 2.5σ level). Only density for the C2 carboxyl moiety of PSR-4-157 can be observed. The ligand is situated on the surface of the SHV-1 protein adjacent to cymal-6 located distant from the active site. (TIF) [file pone.0085892.s002.tif]
