# Supplementary material for: Penam Sulfones and β-Lactamase Inhibition: SA2-13 and the Importance of the C2 Side Chain Length and Composition
Source: PLoS One. 2014 Jan 16;9(1):e85892. doi: 10.1371/journal.pone.0085892 (PMC3894197; doi:10.1371/journal.pone.0085892)
Supplement: Table S1 — Antimicrobial disc assays. These assays were performed with 10 µg ampicillin and 10 µg inhibitor. The size of the disc and the ampicillin zone size alone is 6 mm. (DOCX) [file pone.0085892.s003.docx]

**Table S1.**

| **Inhibitor** | **Disc Assay Zone (mm) *E. coli* ATCC 35218 (TEM-1)** | **Disc Assay Zone (mm) *E. coli* DH10B PDC-3** | **Disc Assay Zone (mm) *E. coli* DH10B SHV-1** |
| --- | --- | --- | --- |
|  |  |  |  |
|  |  |  |  |
| **SA2-13** | 14 | 6 | 6 |
| **PSR-4-157** | 11 | 6 | 6 |
| **PSR-3-226** | 12 | 12 | 6 |
| **PSR-4-155** | 13 | 6 | 6 |
| **tazobactam** | 21 | 14 | 6 |
|  |  |  |  |
